# Some Problems in Infection and Its Control

**Published:** 1913-02

**Authors:** 


### Some Problems in Infection and Its Control.

Simon Flexner (Hurley lecture, reported in *British Medical Journal*) chose poliomyelitis as his general theme because it had claimed so much of his attention during the past few years and because it illustrated admirably certain truths to which he wished to call attention. He reviews the experimental investigation of the disease, routes of infection, pathological effects on the spinal cord and brain, the infectious agent of poliomyelitis and filterable viruses. It is of great interest to determine the correspondence between the general data reviewed and the special facts of poliomyelitis which have been shown to arise in consequence of an invasion of the nervous tissue by an ultramicroscopic or filtrable virus. The virus stands midway between the finest and coarsest examples. It is highly resistant to drying, light and chemical action. In dust it survives weeks and months; in diffuse daylight, indefinitely, and it resists the action of pure glycerin and carbolic acid in 0.5 per cent solution for many months. Recovery from poliomyelitis is attended and produced by an immunization of the body. Active immunity can be achieved by means of the living virus, but thus far no immunizing effect has been produced with the dead virus. After discussing at length the methods of exit and entrance of the virus of poliomyelitis, Dr. Flexner expresses the view that the nasal mucous membrane is the site both of ingress and of egress of the virus of poliomyelitis in man. Support for this view is found also in the study of microscopic changes in the meninges and the central nervous tissues. The virus survives in the dry state and in one instance has been detected in the sweepings of a room occupied by a patient suffering from the disease. The distribution of the virus in coughing and speaking is readily accomplished, and by this means both active cases and passive carriers may conceivably be produced. It should be remembered that we possess no means of discovering the virus except by animal inoculation. Should the experimental results arising from the inoculation of the secretions of the nose and throat of such healthy carriers be confirmed the evidence for the mode of infection as outlined would be complete. Experiments showing the probability of insects and animals acting as carriers of poliomyelitis are reviewed, attention is called to the identity of sporadic and epidemic cases and observations on pneumococcus meningitis and the anti-pneumococcus serum related. As to treatment, the immune serum has thus far acted best when it is injected into the subdural space on several successive days. Such brilliant success has been recently recorded in respect to the specific chemical therapeutics of infection that an effort is being made to attack the problem from this quarter. The point of departure which has been adopted is the drug hexamenthylenamine, which possesses a degree of antiseptic action in the body and is known to be secreted in the cerebro-spinal fluid. When the drug is administered by mouth it can be detected in the fluid in a short time. When inoculations of the virus and administration of the drug are begun together and the administration continued for some days afterward, the development of the paralysis is sometimes, but not always, averted. Hexamenthylenamine lends itself to modifications by the addition of still other antiseptic groups to its molecule. None of this series of drugs is without some injurious effect upon the sensitive and vital organs of the body. Experimentation has already eliminated some of the objectionable features of the drug and improved the valuable features of the drugs so that they exert their action but little upon the organs and severely upon the parasites. When this effect is satisfactorily accomplished, the victory will be won.—*New York Medical Record.*

